# Shedding light on the effects of conflict management training: A multi-rater assessment shines a spotlight on medical students’ skills

**DOI:** 10.1371/journal.pone.0325499

**Published:** 2025-06-20

**Authors:** Fatemeh Mohseni, Aeen Mohammadi, Nasim Khajavirad, Kamal Basiri, Larry Gruppen, Mahboobeh Khabaz Mafinejad

**Affiliations:** 1 Education Development Center, Gerash University of Medical Sciences, Gerash, Iran; 2 Department of Medical Education, Tehran University of Medical Sciences, Tehran, Iran; 3 Department of E-Learning in Medical Education, Center of Excellence for E-learning in Medical Education, School of Medicine, Tehran University of Medical Sciences, Tehran, Iran; 4 Department of Internal Medicine, Imam Khomeini Hospital Complex, Tehran University of Medical Sciences, Tehran, Iran; 5 Department of Emergency Medicine, Prehospital and Hospital Emergency Research Center, Tehran University of Medical Sciences, Tehran, Iran; 6 Department of Learning Health Sciences, University of Michigan, Ann Arbor, Michigan, United States of America; 7 Department of Medical Education, Health Professions Education Research Center, Education Development Center, Tehran University of Medical Sciences, Tehran, Iran; Birjand University of Medical Sciences, IRAN, ISLAMIC REPUBLIC OF

## Abstract

**Background:**

Medical students are repeatedly exposed to challenging situations while working with healthcare teams, so acquiring conflict management skills is necessary. This study aimed to investigate the effect of an educational intervention on the conflict management skills of medical students using self- and observer-assessment.

**Methods:**

This educational intervention with a pre-and post-test design was conducted in 2022–2023. Second-year medical students of Tehran University of Medical Sciences volunteered to participate in a randomized study with a control group. The participants were divided into two intervention (12 groups of 4 each, n = 48) and control groups (12 groups of 4 each, n = 48). The intervention group was educated based on the Fogg model, and the control group was trained using conventional method. Student conflict management skills were evaluated using a self-assessment checklist and observer-assessment.

**Results:**

The findings of observer-assessment revealed that the post-test rating in the intervention group was significantly higher than the control group, while the pre-test score in the two groups did not indicate a significant difference (P = 0.03; ES = 0.44 and P = 0.30; ES = 0.18, respectively). Moreover, the comparison between pre-test and post-test in the two intervention and control groups also showed that the educational intervention significantly increased the mean score of the post-test in both the intervention and control groups (P ≤ 0.001; ES = 0.97 and P ≤ 0.001; ES = 1.34, respectively). The comparison between pre-test and post-test in the two intervention and control groups via self-assessment showed that the skill score increased only in the intervention group (P = 0.02; ES = 0.48 and P = 0.98; ES = 0.004, respectively).

**Conclusion:**

This study found that using the Fogg model in e-learning platforms enhances medical students’ conflict management skills, highlighting the effectiveness of well-designed, creative, and active model-based teaching methods.

## Introduction

Medical graduates must be able to communicate and collaborate to be qualified to work in a healthcare team [1–3]. The Association of American Medical Colleges (AAMC) has emphasized collaboration skills as a critical interpersonal communication competency for a physician [4]. One of the significant barriers to collaboration is conflict among healthcare team members [5]. Conflict refers to the challenge and difference between individuals and teams with opposite priorities, goals, needs, ideas, and values [6]. Although conflict in the workplace is not a new issue, its occurrence is still considered a serious challenge. Evidence shows that workplace conflicts lead to negative consequences such as ineffective teamwork, decreased patient safety and satisfaction, and increased employee turnover and resignation [7–9]. Conflict management skills refer to the ability of healthcare team members to use appropriate and interactive strategies in different conflict situations [10]. Proper conflict management can lead to the formation of innovative ideas and a creative approach to solving challenges, which may lead to strengthening organizational relationships and increasing performance, morale, and commitment in employees [11,12].

Although conflict management skills are considered essential competencies for Medical graduates, no formal training has been incorporated into the curricula. The lack of formal training in conflict management skills leads medical students to primarily learn these skills through trial and error by observing behaviors in the workplace [13,14]. The first step in acquiring conflict management skills is to teach students in a formal curriculum as a member of a healthcare team, which can lead to improved communication and teamwork outcomes [15]. Available evidence suggests that employing the correct methodology during training can yield optimal results. Given the difficulty of modifying ingrained behavior, it is far more advantageous to cultivate appropriate conduct during the initial phases of education [16].

The study conducted by Broukhim et al. revealed that medical students must acquire conflict management skills. Therefore, it is high time for medical schools to prioritize providing opportunities for students to learn these essential skills. It also suggests appropriate approaches to teach conflict management skills [6]. Following the emergence of the Covid-19 pandemic and the emphasis on using e-learning platforms, the effectiveness of educational interventions regarding teaching skills requires more study. To the best of our knowledge, no attempts to investigate the effect of e-learning-based training on the conflict management skills of medical students have been reported. Designing the intervention based on the appropriate framework is crucial to ensure the effectiveness of teaching skills in e-learning platforms, as supported by evidence [17–19]. Fogg behavioral model emphasizes the simultaneous strengthening of the three components of motivation, ability, and prompt [20]. Several studies have used this model as a framework for designing educational interventions in e-learning courses [21–24]. Bardsley, in his study utilizing the Fogg model for intervention design, emphasizes that motivation, ability, and prompts—core components of the model—are crucial factors in improving student learning [23]. Similarly, Mhd Salim’s findings indicate that designing educational courses based on effective models like the Fogg Model enhances learning and significantly influences students’ academic performance in online courses [24]. Furthermore, it is recommended to evaluate the effects of conflict management skills training using performance observation by raters along with self-evaluation methods [19]. The current study aimed to examine the effect of an educational intervention based on the components of the Fogg model as a framework for an e-learning intervention on the conflict management skills of medical students.

## Methods and materials

### Study design and ethical approval

This study is an educational intervention with a pre-and post-test design and a control group conducted from 22nd November 2022–23rd January 2023 (Trial code: IRCT20221005056091N1). The study protocol was approved by the Ethics Committee of the Faculty of Medicine at Tehran University of Medical Sciences (IR.TUMS.MEDICINE.REC.1400.756). At first, the research goals and process were explained to participants, and written informed consent was obtained. All participants were above the legal age of consent, and therefore, parents’ or guardians’ consent was not required. To ensure the confidentiality and anonymity of the participants, the researchers guaranteed that the findings would be used solely for discussion and publication purposes. Participants were informed of their right to withdraw from the study at any time without consequence. All study procedures and methods adhered to the principles outlined in the Declaration of Helsinki.

### Participants

The participants were second-year medical students preparing to enter the physiopathology course at (name of institution). Ninety-six medical students entered the study voluntarily after an invitation. Inclusion criteria were as follows: 1) being a second-year medical student, 2) being interested in participating in the study, 3) not participating in similar courses. Exclusion criteria were as follows: 1) being a guest student in the target schools, or 2) failure to fill out the questionnaire. The participation possibility of male and female students was considered equal to eliminate the effect of gender on the results. The participants were divided into small groups of four students by cluster sampling method and randomly placed into two control (12 groups of 4 each, N = 48) and intervention groups (12 groups of 4 each, N = 48).

At first, the research goals and process were explained to participants, and written informed consent was obtained. The goals, content, and instructors were the same between the intervention and control groups, and only the teaching method in the e-learning platform was different. Teaching was conducted in the control and intervention groups to minimize data contamination, respectively.

### Material preparation

The educational materials, including a handout, five animations, five infographics, four motivational SMS, and four scenarios (Two educational scenarios, one pre-test scenario, and one post-test scenario), were prepared by the research team. An expert panel consisting of eight experts in the field of communication skills was qualitatively reviewed, modified and finalized. Each scenario depicted a conflict situation in a clinical workplace and included four roles so that each role was assigned to one of the four students in each group. Conflict management scenarios were developed and culturally adapted based on the scenarios utilized in the study by Wolfe et al. [25]. The two scenarios used in student training are provided as examples in the S1 Appendix. The compiled material was piloted with four medical students using the same approach as the intervention group.

The educational intervention was designed based on the Fogg model components described in Table 1. According to Fogg model, to improve learning, three components were considered simultaneously. 1) Motivation: To ensure that the student wants to acquire the skill, he/she must have sufficient motivation. Various factors, including pleasure and interest, discomfort, hope, fear and social acceptance or rejection influence student motivation. 2) Ability: To ensure that the student can learn the skill easily. By simplifying the content, skill training is facilitated. 3) Prompt: To ensure that the student is motivated to learn the skill, three formats of prompts include spark, facilitator, and assist signal.

**Table 1:** Educational intervention designed based on the Fogg model.

Motivation	Pleasure/discomfort toward skill acquisition	Synchronous/ Asynchronous	Conflict Management Micro-skills
	• Sending attractive infographics within a three- week period based on simple educational content in a schematic format, including key points in the field of conflict	Asynchronous	• Managing interpersonal and team conflicts, Conflict management styles, Strategic approaches in conflict management, Principled negotiation skills
	**Hope/fear of effects of skill**		
	• Sending motivational mini-lecture to explain the positive effects of using appropriate conflict management styles to the upcoming situations **•** Presenting interesting statistics related to the consequences of not properly managing conflicts by healthcare team members in existing studies	Synchronous	• Conflict management styles, Strategic approaches in conflict management, Principled negotiation skills • Managing interpersonal and team conflicts
	**Social acceptance/social rejection as a result of the presence or lack of skill**		
	**•** Playing a role in simulated situations and trying to reach an agreement in solving scenarios to strengthen the feeling of social acceptance of students in teamwork	Synchronous	• Implementing conflict management strategies in clinical settings, Collaboration and problem-solving in clinical teams
Ability	**Simplify skill acquisition**		
	• Providing 2–3 minutes of micro-learning animations	Asynchronous	• Conflict management styles
	• Providing scenarios and completed negotiation worksheets for effective conflict management to students based on the principle of simplification	Asynchronous	• Principled negotiation skills
Prompt	**Spark as a prompt for attention and motivation**		
	• Presenting evidence-based statistics regarding myths and facts about conflict management strategies	Synchronous	• Strategic approaches in conflict management
	• Viewing a movie clip illustrating a conflict situation in the patient care process, followed by a brief analysis using student polling	Synchronous	• Managing difficult conversations
	**Facilitator as prompt for better understanding**		
	• Presenting conflict management experiences of lecturers in real clinical situations and their effects on their performance and the process of patient care	Synchronous	• Implementing conflict management strategies in clinical settings
	• Showing images and clips regarding the use of verbal and non-verbal communication in conflict situations	Synchronous	• Verbal and non-verbal communication skills
	**Signal as prompt for reminder**		
	• Providing instructors’ feedback while role-playing of students	Synchronous	• Collaboration and problem-solving in clinical teams
	• Sending educational and motivational SMS-based social networks at specified intervals	Asynchronous	• Strategic approaches in conflict management, Collaboration and problem-solving in clinical teams

### Interventions

The educational intervention was conducted using the synchronous-asynchronous e-learning approach. The intervention consisted of two synchronous sessions with an interval of four weeks, during which students studied asynchronous materials. The asynchronous material in the control group was a handout, and in the intervention group, it included four motivational SMS-based social networks, five infographics, and five micro-learning animations that were designed based on the components of the Fogg model, including motivation, ability, and prompt. After completing the first synchronous session, asynchronous materials were sent to the participants at specified intervals (every 2–3 days) through WhatsApp and the learning management system (LMS).

During the first synchronous session, the participants in both groups, before receiving any training, chose one of the pre-test scenario roles to perform the role play and then completed the conflict management skill self-assessment checklist through Google Form™. After studying asynchronous materials during four-week intervals, students participated in a second three-hour synchronous session. The second session was held in three rounds, with 16 participants and four instructors participating in each round.

During this session in the control group, the instructors taught key point through interactive lecturing. In the intervention group, the second three-hour synchronous session was held by presenting a video clip of conflict in healthcare teams and then analyzing the clip with the student participation using polling, interactive discussion, practical exercise using role-playing based on the scenario and providing instructor feedback. The participants of both intervention and control groups were then placed in each of four small groups, and each group acted out the role of the post-test scenario in the presence of one of the instructors. At the end of the session, the students filled out the checklist again.

**Fig 1:**
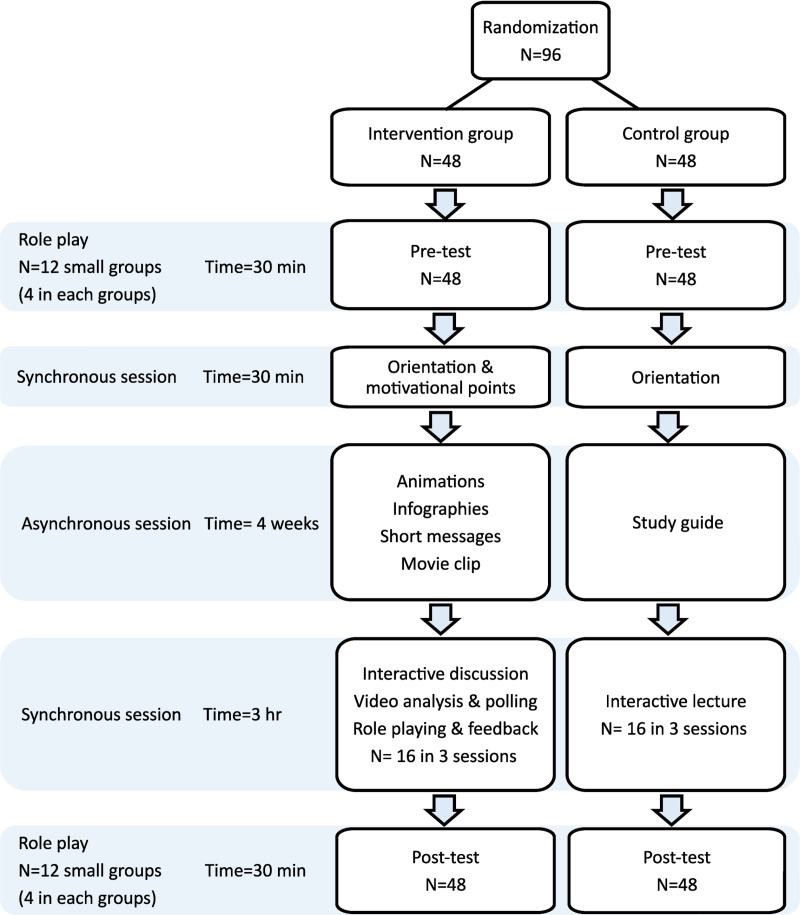
Intervention procedure for both intervention and control groups.

Ability of participants were not only gauged through self-assessment but also evaluated by two independent assessors to assess the student conflict management skills. These assessors are experts in communication skills and possess extensive experience in teaching conflict management. They reviewed recorded videos of role-play activities of each group in both the pre-test and post-test using a checklist. The observers evaluated the recorded videos, which were coded and blind. The educational intervention process for both intervention and control groups is shown in Figure 1.

### Measurements

A researcher-made checklist containing 13 items was used to evaluate conflict management skills in medical students. Exploratory factor analysis revealed four distinct factors: Empathy and Active Listening (4 items), Goal Orientation and Problem-Solving (3 items), Assertiveness and Self-Centeredness (3 items), and Collaborative and Communication Strategies (3 items). Content validity index (CVI) and content validity ratio (CVR) were calculated by surveying nine experts in medical education and communication skills. The researchers performed cognitive interviews with six students to evaluate the face validity of the checklist regarding the clarity and comprehensibility of each item and necessary revisions were made. Cronbach’s alpha coefficient of checklist items was calculated as 0.74. Two trained observers initially independently evaluated the recorded videos of the pilot study. The intraclass correlation coefficient (ICC) was used to evaluate the inter-rater reliability, which was 0.96.

In general, during the study, 48 videos of role playing of small groups of 4 students were recorded (12 videos related to the pre-test of the intervention group, 12 videos related to the post-test of the intervention group, 12 videos related to the pre-test of the control group and 12 videos related to the post-test of the intervention group) and evaluated by two observers independently. The duration of each recorded video was between 15 and 25 minutes. Checklists were completed for each student individually by observers. In total, each observer completed 48 checklists for the intervention group and 48 checklists for the control group. The number of completed self-assessment checklists was equal to 48, in each control and intervention group. The same checklist was used for self-assessment and observer-assessment. Checklist items are provided in the S2 Appendix.

## Statistical analysis

The data were analyzed using IBM SPSS Statistics software (Version 26). The scoring of items 5 and 7 of the checklists was calculated reversely. Descriptive analysis was performed using frequency, percentage, mean, and standard deviation. According to the distribution of data, to compare the variables between the control and intervention groups and also compare the difference in pre-test and post-test skill scores between the two intervention and control groups in the self-assessment section of the Mann-Whitney U test and the observer assessment section of the test, Independent T-test was used. Wilcoxon Signed Rank Test was used to compare the variables within the groups before and after the intervention. The educational effect size was also calculated in comparing groups [26]. A p-value of ≤0.05 was considered statistically significant.

### Results

Ninety-six second-year medical students entered the study voluntarily. The average age of the students was 19.58 (±0.74) in the intervention group and 19.71 (±0.58) in the control group, and there was no significant difference (P = 0.334).

### Self-assessment

The pre-test and post-test skill ratings showed no significant difference between the two groups (P = 0.07 and P = 0.72, respectively). The comparison between the pre-test and post-test in each intervention and control group showed that the mean scores increased, but this increase was significant only in the intervention group (P = 0.02; ES = 0.48 and P = 0.98; ES = 0.004, respectively). Comparison of pre-test and post-test skill score differences between intervention and control groups showed no significant difference (P = 0.08) (Table 2).

**Table 2:** Comparison of pre-test and post-test self-assessment skill scores in intervention and control groups.

Variables	Test	Control group (N = 48)		Intervention group (N = 48)		P-value^a^	Effect size^c^
		Mean rank	Mean (SD)	Mean rank	Mean (SD)		
Self-assessment	Pre-test	50.49	10.08 (2.17)	48.51	9.86 (2.30)	0.726	0.07
	Post-test	44.48	9.92 (2.63)	54.52	10.59 (2.16)	0.074	0.30
		P-value^b^: 0.984, Effect size^c^: 0.004		P-value^b^: 0.023^*^, Effect size^c^: 0.48			
Difference Between pre and post-test for self-assessment	**Groups**	**Mean rank**	**P-value** ^a^	**Effect size** ^**c**^			
	Control group	44.52	0.08	0.30			
		Intervention group	54.48				

^a^Mann‑Whitney U

^b^Wilcoxon Signed Rank Test

^c^Cohen’s effect size

A p-value of <0.05 was considered significant.

### Observer-assessment

The post-test in the intervention group was significantly higher than the control group, while the pre-test score in the two groups did not indicate a significant difference (P = 0.03; ES = 0.44 and P = 0.30; ES = 0.18, respectively). The comparison between the pre-test and post-test in each intervention and control group indicated that the mean score of the post-test in both the intervention and control groups increased significantly (P ≤ 0.001; ES = 0.97 and P ≤ 0.001; ES = 1.34, respectively). Comparison of the difference between pre-test and post-test scores between intervention and control groups showed that educational intervention significantly affected scores (P = 0.03; ES = 0.44) (Table 3).

**Table 3:** Comparison of pre and post-test observer assessment skill scores in intervention and control groups.

Variables	Test	Control group (N = 48)		Intervention group (N = 48)		P-value^a^	Effect size^d^
		Mean rank	Mean (SD)	Mean rank	Mean (SD)		
Observer assessment	Pre-test	51.96	6.69 (2.54)	46.10	6.14 (2.47)	0.304	0.18
	Post-test	42.46	8.88 (2.39)	54.30	9.70 (2.41)	0.037^*^	0.44
		P-value^b^: P ≤ 0.001^*^, Effect size^d^: 0.97		P-value^b^: P ≤ 0.001^*^, Effect size^d^: 1.34			
Difference Between pre and post-test for observer assessment	**Groups**	**Mean difference (SD)**	**P-value** ^c^	**Effect size** ^**d**^			
	Control group	2.25 (2.97)	0.033^*^	0.44			
		Intervention group	3.56 (3.00)				

^a^Mann‑Whitney U

^b^Wilcoxon Signed Rank Test

^c^T-test; ^d^Cohen’s effect size

A p-value of <0.05 was considered significant.

## Discussion

This study evaluated the conflict management skills of second-year medical students of TUMS before and after the educational intervention in the context of electronic learning based on the Fogg model. The findings revealed a significant difference between the pre-test and post-test scores of conflict management skills in both the intervention and control groups. Furthermore, the difference between the pre-test and post-test scores of the intervention group was significantly higher than the control group, and an acceptable effect size was obtained in both groups. Undeniably, the findings of this research confirmed the hypothesis about the effect of educational intervention based on the Fogg model on improving medical students’ conflict management skills. The findings of Cochran et al.’s study showed that conflict management training positively and significantly affects the skills of medical students. Despite these results, the authors expressed concerns about the actual improvement of students’ conflict management skills, as their assessment was solely based on self-reports in the study [10]. This research aimed to ensure the credibility of the findings by incorporating not only self-assessment by students but also the input of two seasoned experts in communication skills who possess extensive experience in teaching and evaluating conflict resolution. Moreover, the systematic review findings highlighted that when assessing the capabilities of physicians, the outcomes derived from evaluations conducted by an external observer are deemed to be more dependable [27].

In some studies, due to the lack of access to valid and reliable tools, qualitative methods such as semi-structured interviews have been used to investigate the impact of the conflict management training intervention [28]. In the current study, a researcher-made checklist was designed and validated to evaluate the conflict management skills of medical students, which can also be used in other studies to evaluate conflict management skills. Access to appropriate tools for assessment can help evaluators have a more accurate insight into the expected performance of students [29–31].

Based on the findings of this study, it is possible to improve students’ ability by simultaneously strengthening the three components of Fogg model, including motivation, ability, and prompt. In a retrospective study with an interprofessional education approach, findings showed that students with motivation and a positive attitude to conflict management show a higher level of learning and a desire to transfer the learned skills to the real environment [32]. According to Colquitt, a positive correlation exists between motivation to learn skills and the transfer of learning after training [33]. The present study focuses on enhancing students’ conflict management skills by combining motivation and ability. We break down the training into manageable steps and simplify the process while also boosting motivation through engaging educational materials like animations, clips, and infographics. Additionally, this research sends short motivational messages based on the Fogg model to guide students toward further skill improvement. Moreover, the evidence shows that in today’s fast-paced world, where people are faced with numerous educational resources, using an e-learning approach based on micro-learning with an emphasis on creating motivation for studying is of great interest [34,35]. The materials should be well-designed regardless of whether they can include clips, infographics, or animations. Alqurashi emphasizes that micro-learning should be designed based on accurate and correct principles [36]. The results of this study indicated that the design of micro-learning materials and raising the level of interaction through sending notifications [34] have effectively improved students’ conflict management skills.

The present findings related to the student self-assessment of conflict management skills showed that the difference in the average scores of students’ pre-test and post-test skills in the intervention group was statistically significant, and the effect size was acceptable, while in the control group, no statistically significant difference was found. These findings are consistent with the results of previous studies that indicate that interventions designed based on behavioral models, such as Fogg model, can effectively improve students’ abilities [37]. Comparing the average scores of students’ self-assessments with the scores of the observers’ assessments presented that the pre-test scores were significantly higher than those of the observer assessments. The results obtained in the study of Lifches et al. are similar to the present results. After conducting a standardized patient-assisted conflict management training course, they simultaneously used self-evaluation and evaluation by SPs and faculty members to check the effectiveness of the course. The results showed that students’ self-evaluation grades have a weak correlation with the scores of SPs and faculty members. According to Lifchez, since the observers were experts in this field, they probably recognized clues of weak interactions and collaboration in the students’ performance that they could not to recognize [38]. Furthermore, based on the obtained findings, it can be interpreted as a possibility that students exaggerate in self-assessment, specifically in evaluating their characteristics, traits, and abilities. In this regard, it has been mentioned in other studies that most medical students tend to show themselves as desirable individuals [37,39]. Based on this, there is a possibility that the students of this study also suffered from this bias. Conversely, the elevated mean scores on the initial assessment within the self-evaluation may be linked to the phenomenon known as the Unskilled-and-Unaware pattern. This pattern suggests that individuals tend to overestimate their abilities in a particular field where they lack expertise, but this overestimation diminishes as they gain proficiency in that field [40]. On the other hand, learning conflict management skills through role-playing in simulated conflict situations can help students become more aware of their real abilities and be more cautious in evaluating their skills and with more knowledge in the post-test self-assessment.

One of the strengths of this study was the simultaneous assessment of conflict management skills through student self-assessment and observer-assessment. In addition, the evaluation of students’ conflict management skills was done by observers independently and without attending the role-playing session. This issue can control the effect of the presence of observers on students’ performance. Moreover, including equal participants of both sexes in the study and eliminating the effect of gender are other strengths of the study. The results of some studies have shown that the limitation of the duration of role-playing can affect the representation of some communication skills by creating a ceiling effect [41]. Moreover, the present study allocated ample time for role-playing activities. Following a suitable model, we designed this study as a randomized controlled trial, incorporating both pre-test and post-test evaluations. However, the study faced some limitations. The voluntary participation of students may impact the generalizability of the obtained findings. Undeniably, it is entirely possible that the students who participated in the study were initially more motivated and possessed unique personality traits. Longitudinal studies are suggested to follow up on the effectiveness of the educational method on students’ skills in the workplace and with repeated encounters. Another limitation was the familiarity of students with each other and their grouping to role play in the scenario, which can cause more cautious behaviors in simulated conflict situations.

## Conclusion

This study demonstrated that the conflict management e-learning intervention based on the Fogg model can improve the skills of medical students to manage conflicts in simulated situations based on scenarios. This approach creates the insight for medical educators that effective training of skills can be implemented with appropriate design and the use of creative, combined, and model-based methods and active methods in the e-learning approach. Moreover, measuring students’ performance using self-assessment and observer assessment can help the reliability and validity of the data. It is suggested that longitudinal studies be conducted to follow the effectiveness of the educational intervention based on the Fogg model and the effect on students’ skills in the workplace.

## Supporting information

S1 Appendix Conflict management scenarios. (DOCX)

S2 Appendix Items to evaluate students’ conflict management skills. (DOCX)
