# Supplementary material for: Shedding light on the effects of conflict management training: A multi-rater assessment shines a spotlight on medical students’ skills
Source: PLoS One. 2025 Jun 20;20(6):e0325499. doi: 10.1371/journal.pone.0325499 (PMC12180622; doi:10.1371/journal.pone.0325499)
Supplement: S1 Appendix — (DOCX) [file pone.0325499.s001.docx]

**S1 Appendix. Conflict Management Scenarios**

**Scenario 1: Conflict in the Process of Patient Prescription and Care Among Colleagues**

Dr. Ahmadi, a pediatric resident, is responsible for examining patients in the clinic. One of her patients, a 1-month-old infant girl, presents with a wound near her eye. The lesion appeared shortly after birth and has grown in size over the past two weeks. Upon examination and review of the patient’s history, Dr. Ahmadi concludes that the lesion is likely an infantile hemangioma.

After reviewing the available evidence and research, she determines that initiating treatment with propranolol is a supported approach, particularly for lesions near the eyes. However, Dr. Bidari, a senior attending physician with more experience, has never used propranolol for treating infantile hemangiomas. He argues that these lesions typically resolve spontaneously by the age of one without intervention.

Dr. Ahmadi presents the evidence she has gathered to Dr. Bidari and suggests either initiating treatment with propranolol or referring the patient to another specialist. However, Dr. Bidari expresses concerns about using propranolol in infants and refuses both to treat the patient and to refer her to another specialist. Despite this, Dr. Ahmadi believes the lesion is progressing in a way that could threaten the patient’s vision. Consequently, she reports Dr. Bidari’s decision to the hospital’s safety committee.

Following this incident, Dr. Bidari informs the residency program director that he will no longer collaborate with Dr. Ahmadi.

**Task:**
As members of the hospital’s patient safety committee, your group must decide how to address this issue.
Demonstrate how you would interact with other group members (the resident, attending physician, hospital’s educational deputy, and the safety committee chairperson) to resolve the conflict.

Your decision must be based on group consensus—each member must agree on the proposed plan or course of action.

**Note:**
Dear students, please keep in mind that the objective of this scenario and role-play is to manage and resolve the presented conflict. Scientific and ethical discussions are not the focus here.

**Scenario 2: Conflict in Supervisory Oversight of a Student’s Performance**

In the emergency department, an intern encounters a patient with nephrotic syndrome who has come for a weekly injection of albumin and furosemide. The intern presents the patient to the attending physician, who states that they will examine the patient thoroughly later and instructs the intern to proceed with the prescribed orders in the meantime.

The intern follows the documented instructions in the patient’s file but forgets to ensure that heparin is administered to flush the patient’s subcutaneous port (a central venous access device). After completing the injections, the nurse disconnects the central line but also forgets to flush it with heparin, as there were no explicit orders for this. As a result, the central venous port becomes clotted, making it unusable for the next medication administration.

The patient’s mother, frustrated by the situation, demands to speak with both the intern and the attending physician. She wants to understand how such an error occurred, prompting the hospital to hold a meeting involving all individuals responsible for the incident.

**Task:**
Decide how to address this situation. As a group member, demonstrate how you would interact with other individuals involved (intern, attending physician, nurse, and the safety committee chairperson) to resolve the conflict.

**Note:**
Dear students, please remember that the purpose of this scenario and role-play is to manage and resolve the presented conflict. Scientific and ethical discussions are not the focus here.
