# Supplementary material for: Shedding light on the effects of conflict management training: A multi-rater assessment shines a spotlight on medical students’ skills
Source: PLoS One. 2025 Jun 20;20(6):e0325499. doi: 10.1371/journal.pone.0325499 (PMC12180622; doi:10.1371/journal.pone.0325499)
Supplement: S2 Appendix — (DOCX) [file pone.0325499.s002.docx]

**S2 Appendix.** Items to evaluate students’ conflict management skills

| Items | Yes | No |
| --- | --- | --- |
| 1. The subject actively listened to the other party’s reasons and words. |  |  |
| 1. The subject defined their goals. |  |  |
| 1. The subject maintained appropriate eye contact for effective interaction. |  |  |
| 1. The subject used non-verbal communication (e.g., open hands, open face) to convey their thoughts and feelings. |  |  |
| 1. The subject had a certain bias towards realizing their own interests. |  |  |
| 1. The subject took a firm position and remained steadfast in their principles. |  |  |
| 1. The subject imposed their views on the other side, not allowing them the opportunity to present their views. |  |  |
| 1. The subject utilized appropriate conflict management strategies (e.g., collaborating and problem-solving) in the given situation. |  |  |
| 1. The subject avoided hurting the feelings of the other party. |  |  |
| 1. The subject identified the points of agreement between the parties. |  |  |
| 1. The subject proposed effective solutions to solve and manage the conflict. |  |  |
| 1. The subject focused on achieving a mutually beneficial, win-win solution. |  |  |
| 1. The subject took responsibility for reaching an agreement. |  |  |
